# Early diagnostic value of Bcl-3 localization in colorectal cancer

**DOI:** 10.1186/s12885-015-1342-6

**Published:** 2015-05-01

**Authors:** Karunakar Saamarthy, Sofie Björner, Martin Johansson, Göran Landberg, Ramin Massoumi, Karin Jirström, Katarzyna Chmielarska Masoumi

**Affiliations:** 1 Department of Laboratory Medicine, Translational Cancer Research, Division of Molecular Tumour Pathology, Lund University, Medicon Village, Building 404:A3, 223 83 Lund, Sweden; 2 Center for Molecular Pathology, Department of Laboratory Medicine, Lund University, Lund, Skåne University Hospital, 205 02, Malmö, Sweden; 3 Sahlgrenska Cancer Centre, University of Gothenburg, 405 30, Gothenburg, Sweden; 4 Department of Clinical Sciences, Division of Oncology and Pathology, Lund University, Skåne University Hospital, 221 85, Lund, Sweden

**Keywords:** Colorectal cancer, Bcl-3, Nuclear fraction

## Abstract

**Background:**

B-cell leukemia 3 (Bcl-3) is a member of the inhibitor of κB family, which regulates a wide range of biological processes by functioning as a transcriptional activator or as a repressor of target genes. Elevated expression, sustained nuclear accumulation, and uncontrolled activation of Bcl-3 causes increased cellular proliferation or survival, dependent on the tissue and type of stimuli.

**Methods:**

We retrospectively reviewed patients who were diagnosed with colorectal cancer at Skåne University Hospital in Malmö between 1st of January 1990 and 31st of December 1991. Bcl-3 localization in colorectal cancer was assessed by immunohistochemistry on tissue microarray and freshly isolated colon from patients. Correlation between Bcl-3 localization and clinicopathological parameters of the cohort were evaluated using the Spearman rank-order correlation coefficient. In addition, Bcl-3 expression and localization in colon adenocarcinoma cells were analysed by western blot, immunohistochemistry and subcellular fractionation separately.

**Results:**

We found that Bcl-3 was mainly localized in the cytoplasm in the tumour tissue isolated from colon cancer patients. Normal colon samples from the same patients showed Bcl-3 localization in the nucleus. In three out of six colon cancer cell lines, we detected elevated levels of Bcl-3. In these cell lines Bcl-3 was accumulated in the cytosol. We confirmed these findings by analysing Bcl-3 localization in a colon tissue micro array consisting of 270 cases. In these samples Bcl-3 localization correlated with the proliferation marker Ki-67, but not with the apoptotic marker Caspase 3.

**Conclusion:**

These findings indicate that analysis of the subcellular localization of Bcl-3 could be a potential-early diagnostic marker in colon cancer.

## Background

Colorectal cancer (CRC) is the third most common fatal cancer and its incidence increases with age. Early detection, adequate surgical excision and optimal adjuvant treatment are of critical importance for outcome [1]. In most cases, CRC has a long incubation time, and as the transformation from healthy tissue to cancerous tissue could take a long time there is an increased risk for elderly patients [2]. The clinical stage of the cancer is determined using the TNM staging system, where T stands for tumour stage, N denotes nodal stage and M metastatic stage. N-stage discriminates between stage II and stage III disease, whereas positive M-status automatically places the patient into the stage IV category [3].

B-cell leukemia 3 (Bcl-3) was originally identified as a gene involved in the recurring chromosomal translocation t (14; 19), which is found in patients with chronic lymphocytic leukemia [4]. Bcl-3 is largely a nuclear protein and an atypical inhibitor of κB (IκB) that does not sequester nuclear factor kappa-light-chain-enhancer of activated B cells (NF-κB) in the cytoplasm. Instead, Bcl-3 binds to target genes by binding directly to promoter-bound homodimers of nuclear factor NF-kappa-B p50 subunit (p50) or nuclear factor NF-kappa-B p52 subunit (p52) and subsequently induces either transactivation or repression of target gene [5]. Bcl-3 together with p50 can form an auto-regulatory loop and thereby represses its own transcription levels [6]. Since the discovery that Bcl-3 is an oncogene involved in leukemia, we and others have determined that Bcl-3 also functions as an oncogene in solid tumours including breast [7], nasopharyngeal [8], endometrial carcinomas [9], colorectal cancer [10], and prostate cancer [11].

High Bcl-3 expression has also been detected in melanoma and other types of skin cancer, such as basal cell carcinoma and cylindroma [12-14]. Recently, Cogswell *et al.* [7] showed that breast tumour tissue contained significantly more nuclear Bcl-3 compared to the adjacent tissue. Later, it was shown that mice overexpressing NF-κB family member c-Rel proto-oncogene protein (c-Rel) under the MMTV promoter developed mammary gland tumours that had elevated levels of p50, p52, nuclear factor NF-kappa-B p65 subunit (p65), V-Rel avian reticuloendotheliosis viral oncogene homolog B (RelB) and Bcl-3 in the nucleus [15]. Another oncogenic property of Bcl-3 is the ability to promote cell survival in cancer cells [16]. The tumour suppressor protein p53 (p53) is an important regulator of cell survival and apoptosis. DNA damage can induce p53 activity and subsequently trigger the apoptotic programme. Under normal conditions the E3-ligase mouse double minute protein 2 (Mdm2) ubiquitinates p53, leading to degradation in the proteasome. The Mdm2 promoter contains a NF-κB binding site and Bcl-3 is required to activate its expression. When Bcl-3 is overexpressed in breast cancer cell lines, it can inhibit DNA damage-induced p53 activity, resulting in increased survival [17]. Bcl-3 can be localized both in the cytosol or nuclei depending on the cell type or stimuli. One example is the activation of keratinocytes with 12-*O*-tetradecanoylphorbol-13-acetate (TPA), which induces the nuclear translocation of Bcl-3. The inhibitory action of Bcl-3 nuclear translocation is executed by the tumour suppressor protein cylindromatosis (CYLD), which can bind directly to Bcl-3 and prevent its nuclear localization [13].

In the present study, we investigated the early diagnostic value of the subcellular localization of Bcl-3 in CRC. We found that Bcl-3 is mainly localized in the cytoplasm in cancer tissues while nuclear localization of Bcl-3 was detected in non-cancerous tissue. These results were confirmed by analysing Bcl-3 localization in colon cancer specimens and normal colon tissue obtained from 270 patients. Furthermore, we found that Bcl-3 localization correlated with the proliferation marker Ki-67.

## Methods

### Patient material, tissue microarray construction and immunohistochemistry

Colon cancer specimens and normal colon tissue were obtained from 270 patients who were surgically treated for colorectal cancer at Skåne University Hospital in Malmö between 1st January 1990 and 31st December 1991. The cohort has been described in detail previously [18,19]. All specimens were histopathologically re-evaluated on haematoxylin and eosin stained slides and representative areas were marked prior to tissue microarray (TMA) construction. Duplicates of 1.0 mm cores from each tumour, as well as areas containing normal colon tissue from 70 of the included patients, were taken and placed in a recipient block using a semi-automated arraying machine (TMArrayer, Pathology Devices, Westminster, MD, USA). Approval was obtained from the Ethics committee at Lund University (Ref no 445–07) whereby the committee waived the need for consent other than by the option to opt out.

Prior to immunohistochemical staining, 4 μm thick TMA sections were mounted onto glass slides and deparaffinized followed by antigen retrieval using Dako’s PTLink (DAKO, Glostrup, Denmark). Bcl-3 (C-14: sc-185 from Santa Cruz) and cleaved-Caspase-3 (ASP175 from Cell Signaling, Antibody #9661) were detected by immunohistochemistry using Dako’s Autostainerplus with the EnVisionFlex High pH-kit (DAKO). The percentage of positively stained tumour nuclei (%), the intensity of the nuclear staining (scored 0–4), and the intensity of Bcl-3 in the cytoplasm (scored 0–3) were evaluated. All immunohistochemistry scoring was performed by a research associate and a pathologist without knowledge of pathological and clinical data. Spearman’s rank-order correlation coefficient was used to examine the statistical significance of correlations between Bcl-3 expression and other variables. All statistical tests were two sided and the calculations were done in IBM SPSS Statistics version 20.0 (IBM, Armonk, NY).

### Cell culture

All cell lines were obtained from ATCC. Colon cancer cell lines HCT-116 and HT-29 were cultured in McCoy’s 5A medium, while SW-48 and SW-480 cells were maintained in RPMI 1640 medium. LOVO cells were cultured in F-12 Kaighn’s modification media, CACO-2 cells were maintained in DMEM medium and RKO cells in MEM medium. All media were supplemented with 10% fetal bovine serum (Invitrogen), 100 IU/ml penicillin, and 100 μg/ml streptomycin (both Gibco). All cell lines were cultured at 37°C in a humidified atmosphere containing 5% CO_2_.

### Immunoblotting

Cells were placed on ice and the media were aspirated. The cells were washed once with cold phosphate-buffered saline (PBS) and harvested in cold 1 × lysis buffer [50 mM Tris–HCl (pH 7.4), 150 mM NaCl, 1% Triton-100, containing 40 μl/mL complete protease inhibitors (Roche Applied Science)]. Lysates were cleared by centrifugation at 12 000 × g for 10 min at 4°C and the protein content was determined. Equal amounts of protein were electrophoretically separated on 10% SDS/polyacrylamide gels and proteins were transferred onto Immobilion-FL PVDF membranes (Millipore). Membranes were blocked with 5% non-fat milk in PBST for 1 hour at room temperature followed by overnight incubation at 4°C with primary antibodies against Actin (1:40000 MP Biomedicals), α-tubulin (1:4000 Abcam), Lamin B (1:1000 Santa Cruz), Bcl-3 (1:500 Santa Cruz). Primary antibodies were detected with horseradish peroxidase-labelled secondary antibody (1:5000, DAKO). The chemoluminescence was detected with a charge-coupled device camera (Fujifilm).

### Immunofluorescence

Caco-2 cells were cultured on glass cover slips, rinsed twice with PBS and fixed for 4 min using 4% paraformaldehyde in PBS, followed by permeabilization using 0.25% Triton-× 100 in PBS for 10 minutes. After permeabilization, the cells were washed three times in PBS and blocked in 1% Bovine Serum Albumin (BSA) in PBS for 1 hour. Cells were thereafter incubated for 1 h with primary antibody in PBS followed by washing and incubation with Alexa Fluor 546-conjugated antibodies (Molecular Probes) in PBS. Cover slips were mounted on object slides in vectashield with diamidino-2-phenylindole (DAPI) (vector Laboratories). Images were captured using a 40× oil objective and Zeiss LSM 710 confocal system.

### Cell fractionation

In order to separate the cytoplasmic and nuclear fraction of the colon cancer cells they were first lysed using a less stringent Buffer A (10 mM HEPES pH7.9, 10 mM KCl pH7.0, 100 μM EDTA pH 8.0, 1 mM DTT, 0.5 mM PMSF) on ice for 15 minutes followed by centrifugation at 6800xg for 2 minutes. The supernatant containing the cytoplasmic fraction was collected and the pellet washed in Buffer A followed by centrifugation. Washing was repeated four times. Cell pellets were resuspended in more stringent lysis Buffer C (20 mM HEPES pH 7.9, 80 mM NaCl, 1 mM EDTA and EGTA pH 8.0, 1 mM DTT, 1 mM PMSF) and samples were sonicated twice for 20 seconds. Both the cytoplasmic and nuclear fractions were then centrifuged at 14000×g for 20 minutes.

## Results

**Figure 1:**
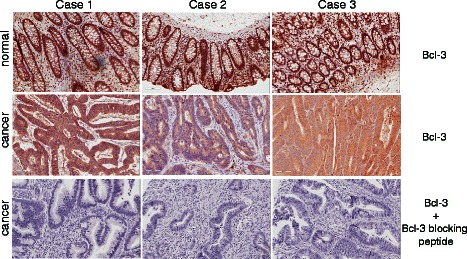
Localization of Bcl-3 in normal colon tissue and in colon cancer. Bcl-3 staining of normal and colorectal tumours from three patients (upper and middle panels). Immunohistochemistry staining of colorectal tumours using an antibody against Bcl-3 with corresponding blocking peptide (p-C14; Santa Cruz) in a concentration ratio 1:5 (Lower panels).

Nuclear localization of Bcl-3 has been reported as an indicator for cell proliferation in different types of cancer [13,15,20]. In the present study, we investigated whether nuclear localization of Bcl-3 in colon cancer has any prognostic value. Surprisingly, we found an accumulation of Bcl-3 in the cytoplasm of colon cancer tissue freshly isolated from three patients. In contrast Bcl-3 was localized in the nucleus of normal tissues (Figure 1). Staining of freshly isolated tumour tissue using an antibody against Bcl-3 with corresponding blocking peptide in a concentration ratio 1:5 demonstrated the specificity of Bcl-3 antibody for immunohistochemistry staining (Figure 1).

**Figure 2:**
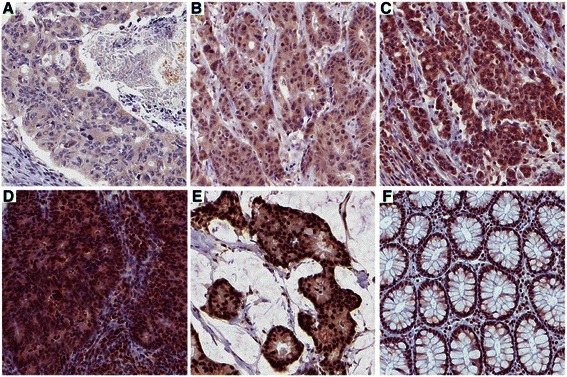
Immunohistological staining of Bcl-3 in colon cancer tissue **(A-E)** and normal colon tissue **(F)**. Nuclear intensity (NI) was scored 0–4, nuclear fraction (NF) was estimated in percent (%) and cytoplasmic intensity (CI) was denoted by scores between 0–3. **(A)** NI = 0, NF = 0, CI = 1. **(B)** NI = 1, NF = 50%, CI = 2. **(C)** NI = 2, NF = 50%, CI = 2. **(D)** NI = 3, NF = 100%, CI = 3. **(E)** NI = 4, NF = 100%, CI = 2. **(F)** Representative image of normal colon tissue, NI = 4, NF = 100%, CI = 1. 20× magnification.

Next, immunohistochemical staining of Bcl-3 was performed in tumours from a patient cohort of 270 CRC patients. Evaluation of the nuclear fraction of Bcl-3 positive cells (estimated in percent), the nuclear intensity (scored 0–4, Figure 2) and the cytoplasmic intensity (scored 0–3, Figure 2) was possible in 264 of 270 (98%) tumour specimens and in 64 of 70 (91%) cases of normal colon tissue (Table 1). The excluded cases were either lost in the staining process or lacked malignant cells in the core biopsy. In 92% of the normal samples 76–100% of all nuclei were positive for Bcl-3 compared to only 33% of the tumour samples (Table 1). This highly significant negative correlation was also observed for the nuclear but not the cytoplasmic intensity of Bcl-3 (Table 1). We found no significant correlation regarding the cytoplasmic intensity of Bcl-3 in normal tissue and tumours (Table 2), whereas a significant correlation between the nuclear fraction of positive cells and the nuclear staining intensity (Spearman’s ρ = 0.512, *p* < 0.001) was detected (Table 2).

**Table 1:** **Bcl3 expression in normal colon tissue and invasive colon cancer**

Variable	Normal tissue	Invasive tumour		
	N = 64 (%)	N = 264 (%)	r	*P* ^†^
Bcl3 nuclear fraction				
0-25%	4 (6)	108 (41)	−0.426	<0.001
26-50%	1 (2)	48 (18)		
51-75%	0 (0)	20 (8)		
76-100%	59 (92)	88 (33)		
Bcl3 nuclear intensity				
Absent	3 (5)	44 (17)	−0.427	<0.001
Weak	0 (0)	17 (6)		
Intermediate/weak	2 (3)	70 (27)		
Intermediate/strong	21 (33)	100 (38)		
Strong	38 (59)	32 (12)		
Bcl3 cytoplasmic intensity				
Absent	2 (3)	7 (3)	−0.045	0.413
Weak	26 (41)	113 (43)		
Intermediate	21 (33)	106 (40)		
Strong	15 (23)	38 (14)		

^†^Correlations were calculated using Spearman’s ρ (two-sided). *P* values were not adjusted for multiple testing.

**Table 2:** **Association between nuclear fraction of Bcl3 and clinicopathological parameters**

	Bcl3 nuclear fraction					
Variable	0-25%	26-50%	51-75%	76-100%	r	*P* ^†^
**N (%)**	108 (41)	48 (18)	20 (8)	88 (33)		
Age						
≤75	59 (55)	30 (63)	10 (50)	48 (55)	0.004	0.948
>75	49 (45)	18 (37)	10 (50)	40 (45)		
Gender						
Female	55 (51)	25 (52)	9 (45)	44 (50)	0.012	0.848
Male	53 (49)	23 (48)	11 (55)	44 (50)		
Tumour stage						
T1	8 (8)	2 (4)	0 (0)	10 (11,5)	−0.097	0.118
T2	22 (20)	15 (33)	4 (21)	26 (30)		
T3	63 (59)	26 (57)	12 (63)	41 (47)		
T4	14 (13)	3 (6)	3 (16)	10 (11,5)		
Node stage						
N0	60 (56)	34 (74)	10 (53)	64 (75)	−0.159	0.011
N1	29 (27)	10 (22)	7 (37)	14 (16)		
N2	18 (17)	2 (4)	2 (10)	8 (9)		
Differentiation grade						
High	9 (8)	2 (4)	0 (0)	10 (11)	−0.045	0.468
Intermediate	67 (62)	37 (77)	15 (75)	55 (63)		
Low	32 (30)	9 (19)	5 (25)	23 (26)		
Ki67 nuclear fraction						
0-25%	8 (8)	6 (13)	0 (0)	3 (4)	0.186	0.003
26-50%	15 (14)	4 (8)	1 (5)	4 (5)		
51-75%	35 (33)	13 (27)	4 (20)	24 (28)		
76-100%	48 (45)	25 (52)	15 (75)	53 (63)		
Bcl3 nuclear intensity						
0 (absent)	44 (41)	0 (0)	0 (0)	0 (0)	0.512	<0.001
1	6 (5)	6 (13)	1 (5)	4 (5)		
2	33 (31)	16 (33)	2 (10)	19 (22)		
3	19 (18)	22 (46)	12 (60)	48 (54)		
4	6 (5)	4 (8)	5 (25)	17 (19)		
Bcl3 cytoplasmic intensity						
0 (absent)	5 (5)	1 (1)	0 (0)	1 (1)	0.190	0.002
1	55 (51)	20 (42)	8 (40)	30 (34)		
2	38 (35)	19 (40)	7 (35)	42 (48)		
3	10 (9)	8 (17)	5 (25)	15 (17)		

^†^Correlations were calculated using Spearman’s ρ (two-sided). *P* values were not adjusted for multiple testing.

**Figure 3:**
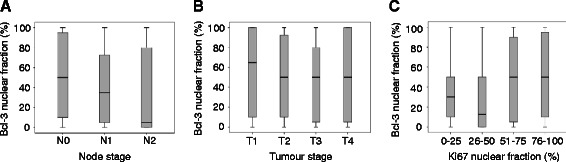
Correlation between nuclear fraction of Bcl-3 (continuous) and **(A)** node stage (r = −0.189, p = 0.002), **(B)** tumour stage (r = −0.100, p = 0.108), and **(C)** nuclear fraction of the proliferation marker Ki67 (r = 0.177, p = 0.004).

**Figure 4:**
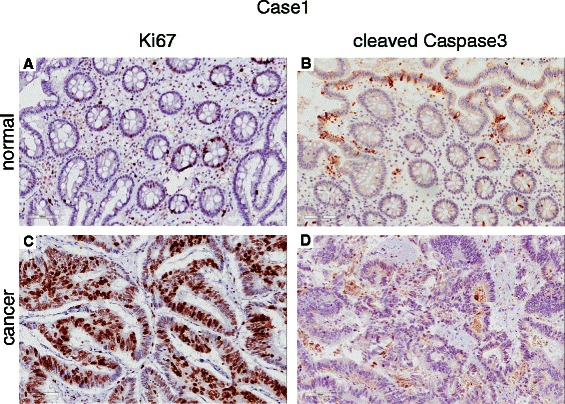
Immunohistochemical sample images with IHC staining representing normal **(A and B)** and colorectal tumours **(C and D)** denoted as Ki67 or cleaved Caspase 3, respectively.

We next investigated the associations between the nuclear fraction of Bcl-3 positive cells and clinicopathological parameters in the CRC tumours (Table 2). A negative correlation with node stage, and a positive correlation with the nuclear fraction of the proliferation marker Ki67, as well as an additional trend toward a negative association with tumour stage, were detected (Figure 3A-C). This result was confirmed by investigating whether cytoplasmic Bcl-3 cancer cells are proliferative or apoptotic cells. Immunohistochemistry staining using freshly isolated CRC showed that Ki-67 but not cleaved Caspase 3 positive cells express Bcl-3 in the cytoplasm (Figure 4). These results suggest that in CRC, the majority of proliferative cancer cells express Bcl-3 in the cytoplasm, whereas non-cancerous colon tissues express Bcl-3 in the nucleus.

**Figure 5:**
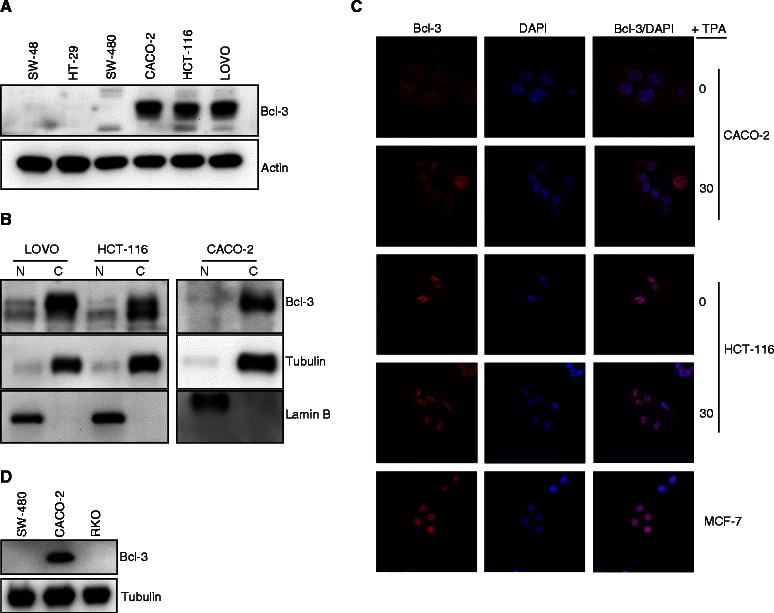
Expression and localization of Bcl-3 in colon cancer cell lines. Total cell lysate of indicated colorectal cell lines was analysed by Western blot for Bcl-3 and actin **(A)**. Subcellular fractions of LOVO, HCT-116 and CACO-2 were prepared. Nuclear and cytosol fractions were analysed by immunoblotting **(B)**. CACO-2, HCT-116, and MCF-7 cells were analysed with immunofluorescence towards Bcl-3. When indicated cells were stimulated with 100 nM TPA. DAPI was used as a nuclear marker. Cells were examined with confocal microscopy **(C)**. Whole cell lysates of the indicated colon cancer cell lines grown in complete medium were subjected to Western blotting **(D)**.

To further evaluate the expression level of Bcl-3 in colon cancer, we prepared total cell lysate from SW-48, HT-29, SW-480, CACO-2, HCT-116 and LOVO and found variation in Bcl-3 expression in these cell lines. High expression of Bcl-3 was detected in CACO-2, HCT-116 and LOVO, while SW-48, HT-29 and SW-480 cells showed lower Bcl-3 expression (Figure 5A). To investigate Bcl-3 localization in colon cancer cell lines, we performed biochemical cell fractionation and immunofluorescence staining. We successfully purified nuclei and cytoplasm using cell fractionation (Figure 5B, see tubulin and lamin B) and found that under basal conditions the majority of Bcl-3 is expressed in the cytoplasm compared with a low level of Bcl-3 in the nucleus (Figure 5B). To confirm this result, we performed immunofluorescence staining and confocal microscopy of Bcl-3 localization in CACO-2, HCT-116, and breast cancer cell line MCF-7 and showed the accumulation of Bcl-3 in the cytoplasm of CACO-2 and HCT-116 but not in MCF-7 cells (Figure 5C). These results suggest that cytoplasmic localization of Bcl-3 is specific to colon cancer compared to breast cancer cell lines. Previously, we could demonstrate re-localization of Bcl-3 in mouse keratinocytes upon TPA treatment [13,21]. To investigate whether we can observe similar translocation of Bcl-3, colon cancer cell line were stimulated with TPA for 30 minutes. No differences in the localization of Bcl-3 comparing TPA stimulated with non-stimulated CACO-2 or HCT-116 cells could be detected (Figure 5C). Can sustained Wnt signalling affect Bcl-3 localization? To test this hypothesis, we used human colon cancer cells RKO, which in contrast to CACO-2 and HCT-116, do not contain mutations in β-catenin or APC [22]. In this cell line the levels of Bcl-3 were undetected (Figure 5D).

## Discussion

The proto-oncogene Bcl-3 is known to be overexpressed and localized in the nuclei of different solid tumours such as breast [7], nasopharyngeal [8], endometrial [9] and prostate cancer [11]. However it is not known whether localization of Bcl-3 in colon cancer is similar to other reported studies, and whether localization of Bcl-3 has any prognostic value in colon cancer. In the present study, we investigated whether localization of Bcl-3 in cancer cells follows the same pattern as in previously reported solid tumours. Surprisingly, we found that in freshly isolated colon biopsies Bcl-3 was localized in the cytoplasm of the cancer tissues, while non-cancerous tissue showed an accumulation of Bcl-3 in the nuclei. These results were confirmed by analysing Bcl-3 localization in a cohort of patients with CRC, in which significant differences in the localization of Bcl-3 between normal tissue and invasive tumours were observed. Cancer cells showing cytoplasmic localization of Bcl-3 were also positive for the proliferation marker Ki67 but not for the apoptotic marker Caspase 3. This suggests that preventing nuclear localization of Bcl-3 in colon cancer cells facilitates proliferation of these cells. In addition, we found cytoplasmic localization of Bcl-3 in three human colon cancer cell lines, confirming the results obtained from our freshly isolated tissues and TMA analysis. A shift from positive Bcl-3 staining in the nucleus to the cytoplasm could indicate the transition of normal colonic epithelia cells into colon cancer.

The role of Bcl-3 in colon cancer is not yet known. A recent retrospective study of 23 patients who underwent surgical resection of CRC showed that nuclear expression of p50, p65 and Bcl-3 is negatively associated with survival [10]. Furthermore, dose-dependent inhibition of COX-2 using NS398 inhibited the expression of Bcl-3 and cyclin D1 in a human colon cancer cell line [23]. In mice, deletion of Bcl-3 protected against chemically-induced colitis compared to wild-type animals. This treatment also resulted in elevated intestinal epithelial cell proliferation in Bcl-3 knockout mice compared with the control, suggesting that Bcl-3 plays a major role in regulating proliferation and sensitivity to chemically induced colitis. Furthermore, Bcl-3 expression in the colon of inflammatory bowel disease (IBD) patients was significantly increased compared to healthy individuals [24].

Bcl-3 can be localized both in the cytoplasm and nucleus dependent on the cell type or stimuli. Two mechanisms for Bcl-3 nuclear transport have been identified. Bcl-3 contains a classical nuclear localization signal (NLS) within its N-terminal domain and thus can be imported into the nucleus [25]. Post-translational modification of Bcl-3 via ubiquitination was also shown to be essential for nuclear localization of Bcl-3. The nuclear translocation of Bcl-3 is blocked by the de-ubiquitination enzyme CYLD via direct interaction and removal of K63-linked polyubiquitin chains from Bcl-3 [13,14]. The mechanism that promotes cytoplasmic retention of Bcl-3, which we observed in normal colonic epithelia cells, is not known. In the nucleus Bcl-3 can initiate or repress the transcription of target genes [11,26]. Future studies are needed to identify the mechanism and signaling pathway that hold Bcl-3 in the cytoplasm of colon cancer cells.

## Conclusions

In summary, we identified a potential role of Bcl-3 in colorectal cancer through its localization in cells and tissue samples. In the cancer tissues or cells Bcl-3 was localized in the cytoplasm, while non-cancerous tissue/cells showed an accumulation of Bcl-3 in the nuclei. This finding implies that Bcl-3 could serve as an early diagnostic marker in colorectal cancer.

## Abbreviations

CRC: Colorectal cancer
Bcl-3: B-cell leukemia 3
IκB: Inhibitor of κB
NF-κB: Nuclear factor kappa-light-chain-enhancer of activated B cells
p50: Nuclear factor NF-kappa-B p50 subunit
p52: Nuclear factor NF-kappa-B p52 subunit
p65: Nuclear factor NF-kappa-B p65 subunit
c-Rel: c-Rel proto-oncogene protein
Rel B: V-Rel avian reticuloendotheliosis viral oncogene homolog B
TPA: 12-*O*-tetradecanoylphorbol-13-acetate
CYLD: Cylindromatosis
TMA: Tissue microarray
PBS: Phosphate-buffered saline
BSA: Bovine serum albumin
DAPI: Diamidino-2-phenylindole
NLS: Nuclear localization signal
IBD: Inflammatory bowel disease
Caspase-3: Cysteine aspartic specific protease 3
p53: Tumour protein p53
Mdm2: Mouse double minute protein 2
Ki-67: Marker of proliferation
COX-2: Cyclooxygenase 2
